# The effects of laser treatment in tendinopathy: a systematic review

**DOI:** 10.1590/1413-78522015230100513

**Published:** 2015

**Authors:** Adelmário Cavalcanti Nogueira, Manoel de Jesus Moura Júnior

**Affiliations:** Universidade Estadual do Piauí, Centro de Ciências da Saúde, Teresina, PI, Brazil, Centro de Ciências da Saúde (CCS) da Universidade Estadual do Piauí, Teresina, PI, Brazil

**Keywords:** Laser therapy, Tendinopathy, Review

## Abstract

Tendons have as main function transmit forces from the muscle to the bones. Tendinopathy is an inflammatory process that occurs in and around the tendon, when these are affected by some injury. Low level laser therapy consists in a local application of a monochromatic, coherent and short wavelength light. Its use began in 60's and since then several benefits for tendon injuries have been reported. The objective of this study is to collect the most recent studies about the use of laser on the tendinopathy treatment. We performed searches on the following electronic databases PubMed, Medline, CAPES journals portal and LILACS. After the analysis, we selected three articles that showed that the use of low-level laser therapy, compared to placebo, is effective in treatment of tendinopathy. Despite the need for more studies about this theme, the low-level laser therapy demonstrates consistent results in the treatment of tendinopathy.

## INTRODUÇÃO

Tendons have as primary function transmit tensile strength from muscles to the bone region^1^ and when affected by some injury, they develop an inflammatory process called tendinopathy, which takes place in and around the tendon.^2^ It is generally characterized by an old and localized pain related to professional or sports activities.^3^ The normal tendon is shiny and white, while the inflamed tendon is gray or brown, soft, thin and fragile.^4^

The incidence of tendinopathy is responsible for about 30% of the number of musculoskeletal^5^ diseases and sedentary lifestyles, coupled with the specific requirements of physical and sports activities contributed to its increase.^6^

Its etiology is unknown, however, extrinsic factors such as the environment, inadequate stretching and warming up and asynchronous muscle action seem to affect its prevalence and recently intrinsic factors such as age and gender have contributed to their apearence.^7^

Achilles tendinopathy is often found in athletes, whose sport involves running and excessive training contributing to its appearance, leading to an increase in the production of fibroblasts and collagen degeneration which can be evidenced by imaging examination.^8^

The clinical prognosis associated with tendon injury and the limited capacity of its regeneration results in an increased interest in the use of approaches to the treatment of tendon.^5^

The physical therapy has been widely used in these cases and consists of exercises and muscle stretching programs, and also other methods such as ultrasound, iontophoresis, deep massage, and low intensity laser therapy.^2^

Low intensity laser therapy consists of a local application of a monochromatic light, coherent and of short wavelengths.^9^ Since 1960 it is used in the treatment of tendinopathy and studies from the 80's, report benefits in a variety of tendon injuries.^10^ It is a generally recommended therapy as a complement to an exercise program in the treatment of tendinopathy, however, when used in isolation, laser does not show to be as effective.^11^

Therefore, this research aims at a systematic review, with the purpose to collect what is latest in low-intensity laser therapy in the treatment of tendinopathy.

## METHODS

A search was performed in the electronic databases PubMed, Medline, Capes Journals Database and Latin American and Caribbean Health Sciences Literature (LILACS), without restrictions regarding the period of publication. We used the following keywords identified in consultation with the DeCS (Descriptors in Health Sciences), "Laser therapy" and "Tendinopathy" combined.

For this review, we only included randomized, double-blind placebo-controlled trials which had as objective evaluating the effectiveness of laser use in humans affected by tendinopathy and that were published in English language. Exclusion criteria were: publication language other than English; meta-analysis articles published prior to 2006.

## RESULTS

From the search performed in the databases we found one hundred and eighty seven articles, of these thirty-one were indexed in PubMed and one hundred and fifty-six articles in Capes Journals Database, being eight of them common to both databases. LILACS and Medline showed no results for the search. After the analysis and filtering of titles, only ten articles were selected from PubMed and six from Capes Journals Database. Of these, four were common to both databases. After the reading the abstracts, only three articles were selected, one present only in Pubmed and two in common, all journals ranked as Qualis B1, totalizing the number of the sample in seventy-nine participants.

The studies had their information summarized on the topics author/year, objectives, sample, frequency of treatment, concomitant interventions, variables analyzed in the study, measurement unit of the variables and results reported.^12^^-^ 14 (Table 1)

**Table 1.** Synthesis of the studies analyzed.

Author/year	Objectives	Sample	Frequency of treatment	Concurrent interventions	Measurement units	Results
Tumilty *et al.*12 (2008)	Verify the effectiveness of low intensity laser associated with eccentric exercises in the treatment of Achilles tendinopathy	21 patients divided in two groups laser and placebo	Use of laser/placebo three times a week during four weeks	Plantar flexion eccentric exercises six sets of 15 repetitions, twice a day, seven days per week for twelve weeks	VISA-A specific for Achilles tendon (0 to 100, 100 being a healthy tendon and 0 incapacitating pain) VAS (0 = no pain and 100 = worst pain imaginable). Measurement of isokinetic muscle strength by Biodex dynamometer. Patients seated at 40° knee flexion, 110° hip flexion. Strength was measured between 20 and 30 dorsiflexion	There was significant improvement of the analyzed criteria. Conclusions about the effectiveness of low-intensity laser therapy cannot be made due to the low statistical power of the study.
Bjordal *et al.*13 (2006)	Verify whether the low intensity laser has no effect anti-inflammatory in Achilles tendinopathy	7 patients With bilateral Achilles tendinopathy Each patient was treated with lase and placebo	One single application	None	Doppler ultrasound to measure intra-tendon blood flow. Concentration of prostaglandin E2 (inflammation quantifiers). The pain spot identified and then compressed until patient ask to stop, which meant pressure switched to pain	The low intensity laser in this dosage can reduce inflammation in activated Achilles tendinitis
Stergioulas *et al.*14 (2008)	low intensity laser associated with eccentric exercises can produce a faster improvement of Achilles tendinopathy	52 athletes divided into two groups: laser+ exercises and laser+placebo	Twelve sessions in eight weeks	Eccentric exercises four times a week for eight weeks	VAS of 100 mm to evaluate morning stiffness, active dorsiflexion, palpation and crackling (0 represents no pain and 100, extreme pain). Goniometer for active dorsiflexion of the ankle	There was an acceleration of the recovery process when laser was associated with an eccentric exercise program

From each study, were retrieved the following information regarding the applicability of laser parameters: wavelength, power, dose, site and time of application. (Table 2)

**Table 2.** Low Intensity Laser parameters used on the studies selected in this review.

Author/year	Wavelength (nm)	Power	Application dose	Local/ time of application de
Tumilty *et al.*12 (2008)	810	100mW/cm2	3J per point e 18J per session	6 standard points in each tendon during 30 s in each point
Bjordal *et al.*13 (2006)	904	20mW/cm2	1.8J per point totalizing 5,4J per session	3 standard points in each tendon during 180 s in each point
Stergioulas *et al.*14 (2008)	820	60mW/cm2	0.9J per point totalizing 5,4J per session	6 standard points in each tendon. The application time was not informed.

nm: nanometers; mW/cm2: miliwatt/square centimeter; J: Joule.

The VISA-A questionnaire is the only valid, reliable, and that has a specific score to measure Achilles tendon condition.^15^ These are questions that assess pain (questions 1-3), function (4-6), and activity (7 and 8). The maximum score for each item from questions one to seven is 10 points, while for question eight it can reach 30 points. Thus, a score of 100 represents a healthy person, whilst zero, presents the worst possible outcome.

The Visual Analogue Scale (VAS) was widely used as the preferred way to evaluate various parameters, such as pain and quality of life due to its easy reproducibility and validity.^16^

The Doppler ultrasound, which measures the amount of blood flow, has the great advantage of being a quick noninvasive and relatively low cost examination.^17^ Patients can easily go do the exam and it is available in most hospitals.^18^

Goniometry finds its relevant place among the orthopedic community, when it comes to measure the range of motion of the joints. It is a quick and easy measurement, but may vary depending on the professional.^19^

## DISCUSSION

After the selection and analysis of searched articles, we noticed that there was a significant improvement of pain in the treatment of Achilles tendinopathy with the application of low intensity laser and in all the studies, application of treatment showed to be effective with consequent relief of symptoms, among them: improvement of pain, of range of motion and motor function. Although Tumilty *et al.* 10 have not performed their study in Achilles tendon tendinopathy, they reported that low-intensity laser therapy promoted reduction of the inflammation in the lateral epicondyle of the elbow due to angiogenesis and increased collagen synthesis, proving the efficacy of this therapy.

The study of Bjordal *et al.* 9 analyzed the anti-inflammatory action of low intensity laser through the concentration of inflammatory markers and found that its use increased blood flow (angiogenesis) in the region and decreased inflammation.

On papers by Tumilty *et al.* 12 and Stergioulas *et al.*,^14^ the laser was used in parallel with the application of an eccentric exercise program, which led to clinical improvement (analyzed factors), while for Stasinopoulos *et al.*,^11^ low-intensity laser also showed positive results in combination with an exercise program in the treatment of tendinopathy, which highlights the importance of the combination of these two therapeutic actions.

Tumilty *et al.*,^10^ after the analysis of twenty-five studies using low-intensity laser in the treatment of several types of tendinopathy, found that in thirteen treated groups no improvement of the symptoms was reported. In their research the variance of these results would be related to factors such as age, gender, type of tendinopathy and the use of different laser intensity parameters, which led to a discrepancy between the results.

Despite the different parameters such as time, power, wavelength and the frequency of laser therapy varied from one study to another, the results were favorable, but the small size of the sample in some studies contributed to a decreased level of confidence of these results.

Therefore, despite the small number of studies found in our review, it became evident the effective contribution of low-intensity laser to the improvement of inflammation by Achilles tendinopathy.

## FINAL CONSIDERATIONS

Generally speaking, all studies have shown that application of low intensity laser was better than placebo in improving the factors analyzed in Achilles tendonitis. It also became evident the contribution of an exercise program combined with the laser in the recovery of injuries.

Assuming the lack of themes and samples associated with the studies found in this review, and the fact that well-known databases were used, it is suggested that further research are made using low-intensity laser therapy in patients with this condition to best assess this therapy. 
